# The Development of Extensive Subcutaneous Emphysema Following Robotic Total Abdominal Colectomy Due to Lynch Syndrome: A Case Report

**DOI:** 10.7759/cureus.57069

**Published:** 2024-03-27

**Authors:** Alexander Garcia, Dylan S Irvine, Lisa Tomasello, Imani Thornton

**Affiliations:** 1 Anesthesiology, HCA Florida Westside Hospital, Plantation, USA; 2 Osteopathic Medicine, Dr. Kiran C. Patel College of Osteopathic Medicine, Nova Southeastern University, Davie, USA; 3 Anesthesiology and Critical Care, HCA Florida Westside Hospital, Plantation, USA

**Keywords:** robotic-assited surgery, medical intensive care unit (micu), airway extubation, critical care anesthesiology, subcutaneous emphysema management

## Abstract

Subcutaneous emphysema, the presence of air in the subcutaneous layer of the skin, is a possible result of surgical, infectious, or spontaneous etiologies. Although usually self-limiting, the development of subcutaneous emphysema in the perioperative period has been associated with delayed extubation and the development of complications such as pneumomediastinum, pneumoperitoneum, and pneumothorax and can worsen clinical outcomes in these patients. Here, we report the case of a 57-year-old male patient who presented to the operating room (OR) for a robotic total colectomy due to Lynch syndrome. The procedure was complicated by the development of diffuse, severe subcutaneous emphysema, which was recognized by palpable crepitus and obscuration of anatomical landmarks during an attempted transversus abdominis plane (TAP) block for pain control prior to patient extubation. The decision was made to leave the patient intubated and managed postoperatively in the ICU, where radiographic and computerized tomography (CT) scans confirmed the severity of subcutaneous emphysema. Hemodynamic and respiratory status were managed in the ICU and on postoperative day 3 the patient passed an endotracheal cuff leak test and was extubated. The patient was transferred to a surgical step-down on postoperative day 7 and following the resolution of ileus and acute kidney injury (AKI), he was discharged from the hospital on postoperative day 17.

## Introduction

Subcutaneous emphysema refers to the presence of air in the subcutaneous layer of the skin, which may develop through infiltration or de novo generation and can occur through surgical, infectious, or spontaneous etiologies [1]. In the perioperative period, the pathophysiology of subcutaneous emphysema development has been attributed to a number of possible causes, including malfunction or disruption of the ventilator circuit, inappropriate closure of the pop-off valve, trauma to the airway, injury to the esophagus during gastric tube placement, chest wall breach in arthroscopic procedures, bowel perforation, complications of central venous access procedures, through the female genital tract during a pelvic examination and following insufflation of the abdomen during laparoscopic or robotic procedures [2]. Although rare, with an estimated incidence of 0.4-2.3% in the general population, approximately 77% of patients who undergo laparoscopic procedures may develop some degree of grossly undetectable subcutaneous emphysema [3,4]. While the majority of these cases are not clinically detectable or significant, the consequences and clinical outcomes depend on the severity of the disease [5]. The development of subcutaneous emphysema has been observed more commonly in males than in females (approximately 70% of cases reported have occurred in male patients), with an average age of approximately 53+/-15 years [3]. Subcutaneous emphysema can result in perioperative complications such as delayed extubation, pneumomediastinum, pneumoperitoneum, and pneumothorax, which can occur with extravasation of air into other body cavities and spaces due to pressure gradients between fascial and anatomic planes [1]. Clinically, subcutaneous emphysema is recognized by palpation of crepitus on physical exam, in addition to possible distention or bloating of the abdomen, neck, face, or chest [6]. In patients with severe presentation, there may be associated hemodynamic or respiratory compromise [4]. Radiographic studies such as x-rays or computed tomography (CT) scans may also be used to identify subcutaneous emphysema and will demonstrate intermittent areas of radiolucency or dark pockets in the subcutaneous layer indicative of gas, respectively [7,8]. Treatment is mostly supportive and involves the management of respiratory and hemodynamic derangements. Once the source is controlled, subcutaneous emphysema will self-resolve in less than 10 days [9]. Patients with severe discomfort or respiratory compromise may benefit from high-concentration oxygen therapy, a well-studied treatment for subcutaneous emphysema [10]. High-concentration oxygen therapy allows diffusion of gas particles and nitrogen washout in these patients and may lead to faster resolution of subcutaneous emphysema [10]. 

## Case presentation

We report the case of a 57-year-old male with Lynch syndrome (hereditary nonpolyposis colorectal cancer syndrome) who presented to the operating room (OR) for a robotic total colectomy due to right-sided colorectal cancer. Preoperative vital signs were blood pressure: 179/113 mmHg, heart rate: 71 bpm, respiratory rate: 14 breaths per minute, temperature: 98.2°F, and oxygen saturation (SpO2): 99%. The preoperative physical exam was within normal limits. Airway examination demonstrated a normal range of motion with a Mallampati class 2 view. Past medical history was significant for Lynch syndrome and left-sided colon cancer. Past surgical history was significant for previous colonoscopy. The patient did not take any medications at home, was a lifetime non-smoker, and had no known drug allergies. 

General anesthesia was induced with 4 mg midazolam, 150 mg propofol, 50 mcg fentanyl, 30 mg rocuronium, and 50 mg lidocaine. A MAC 4 blade was used with direct laryngoscopy and a size 7 endotracheal tube was secured without difficulty at a depth of 22 cm. General anesthesia was maintained with sevoflurane and the patient was ventilated throughout the procedure with synchronized intermittent minute ventilation at a respiratory rate of 14 breaths per minute, tidal volume of 525 mL, and positive end-expiratory pressure (PEEP) of 5. End-tidal carbon dioxide (etCO_2_) levels were maintained at 35-45 throughout the procedure. The patient's intraoperative course was uncomplicated and hemodynamic and respiratory statuses were maintained throughout. 

**Figure 1:**
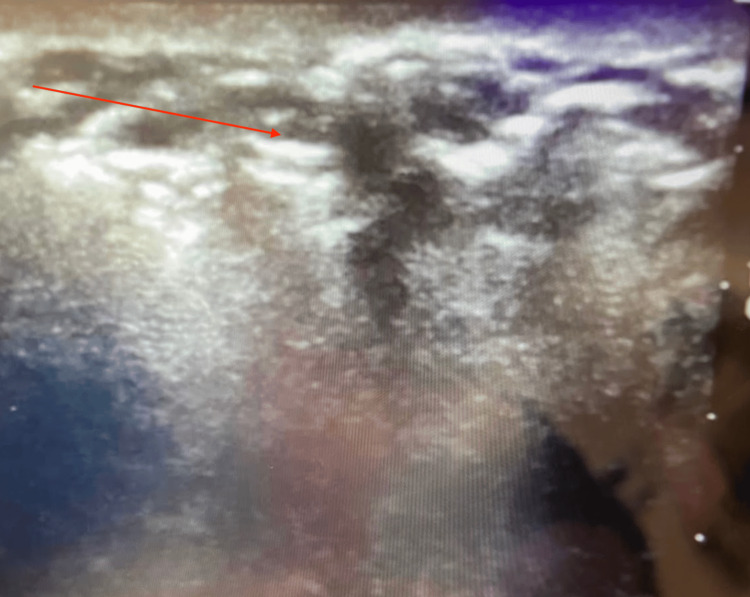
Postoperative, ultrasound-guided TAP block attempt, which revealed obscuration of anatomical landmarks due to extensive subcutaneous air. TAP, transversus abdominis plane

Following the completion of the procedure and abdominal closure, a transversus abdominis plane (TAP) block was attempted with ultrasound guidance prior to extubation to assist with postoperative pain control. While attempting to visualize the landmarks required to perform this block, gross amounts of subcutaneous air were noted and landmarks could not be identified, as demonstrated in Figure 1. Palpation of the patient’s abdomen was significant for extensive crepitus. Upon further examination, extensive, diffuse crepitus was found around the patient’s chest, upper and lower extremities, abdomen, back, head, and neck, and a diagnosis of subcutaneous emphysema was suspected.

Due to the extensive, diffuse crepitus that was palpated on physical exam, and the presence of apparent subcutaneous air visualized on abdominal ultrasound, the anesthesia team decided to keep the patient intubated for airway protection. The patient was transferred from the OR to the intensive care unit (ICU) for close observation and monitoring of hemodynamic and respiratory status. 

**Figure 2:**
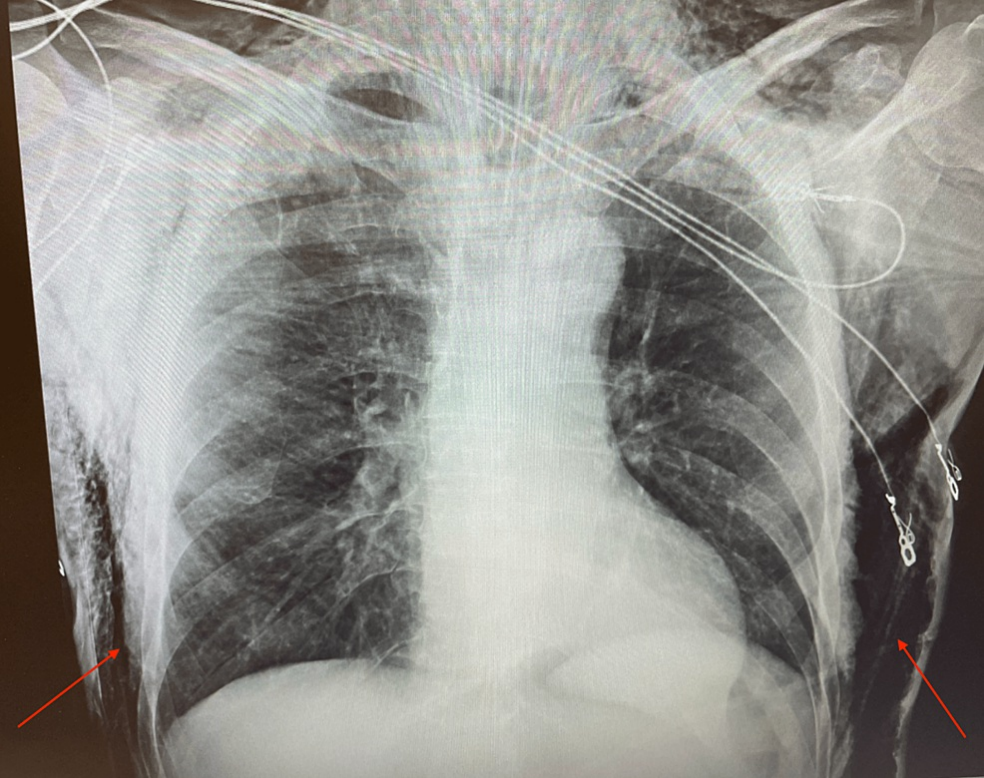
Chest X-ray obtained postoperatively in the ICU, which demonstrated extensive subcutaneous emphysema in the exterior borders of the thoracic walls.

Upon transfer to the ICU, a chest X-ray was performed to confirm proper endotracheal tube placement and confirm the diagnosis of subcutaneous emphysema, as shown in Figure 2. This radiograph demonstrated extensive subcutaneous emphysema and recommended CT scans be completed for further characterization as clinically warranted.

**Figure 3:**
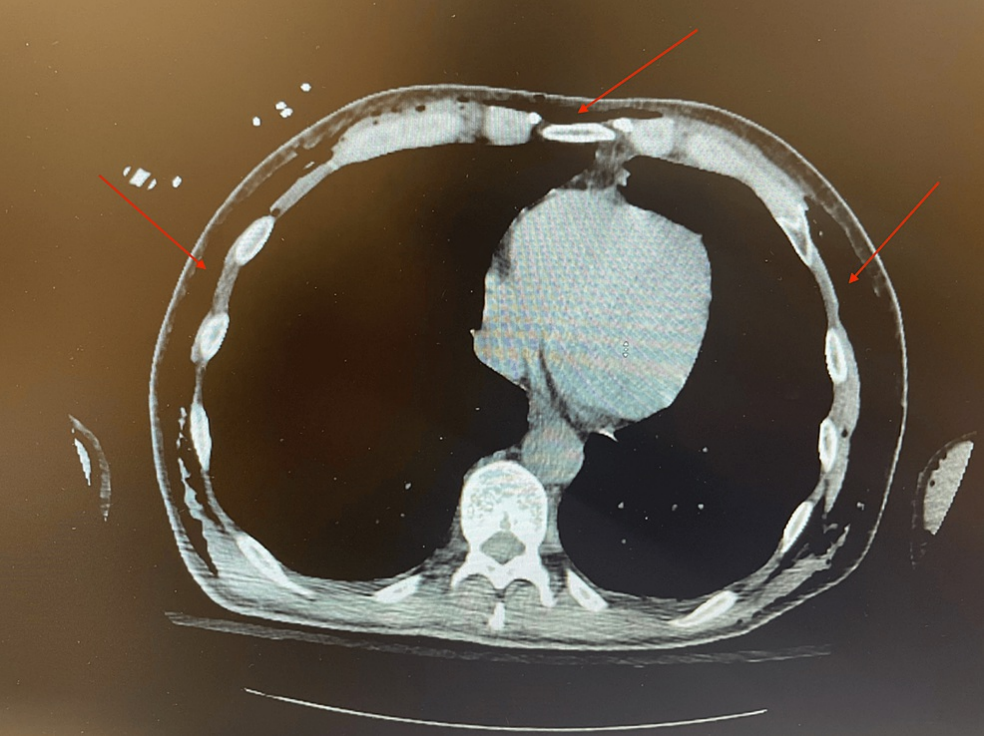
CT scan of the chest obtained postoperatively, which demonstrated extensive soft tissue air in the mediastinum without defects in the tracheal or esophageal wall.

**Figure 4:**
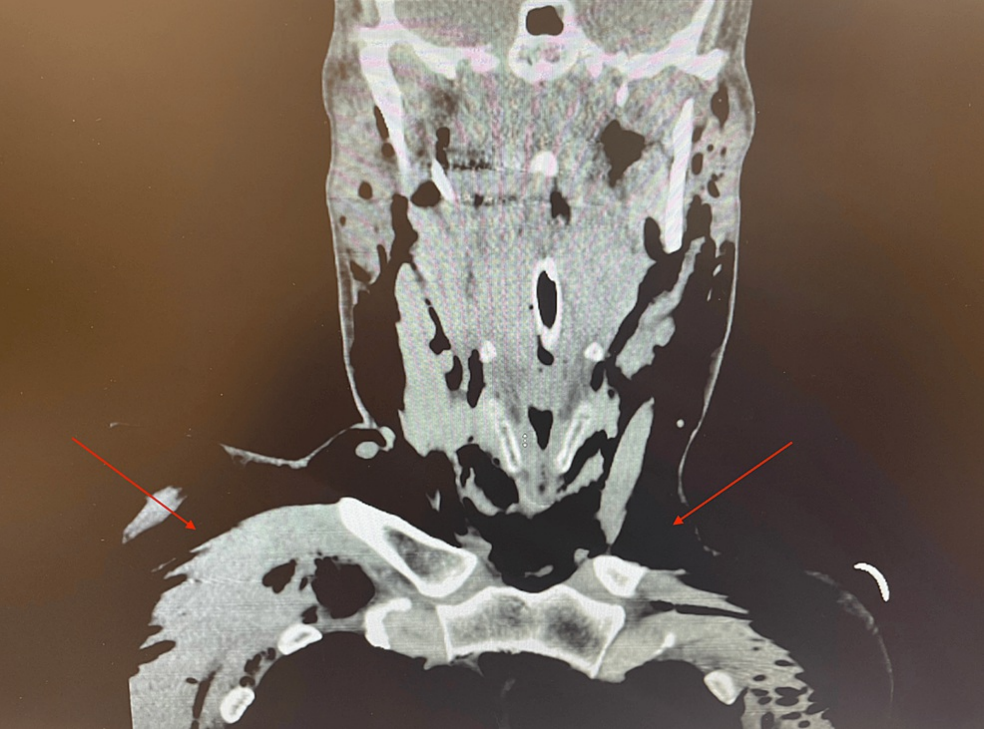
CT scan of the neck obtained postoperatively, which demonstrated extensive soft tissue air without defects in the trachea or esophagus.

A CT scan of the chest demonstrated extensive soft tissue air in the mediastinum and thoracic walls without defects in the tracheal or esophageal walls, as demonstrated in Figure 3. CT scan of the neck similarly demonstrated extensive soft tissue air without defects in the trachea or esophagus, which is shown in Figure 4. 

**Figure 5:**
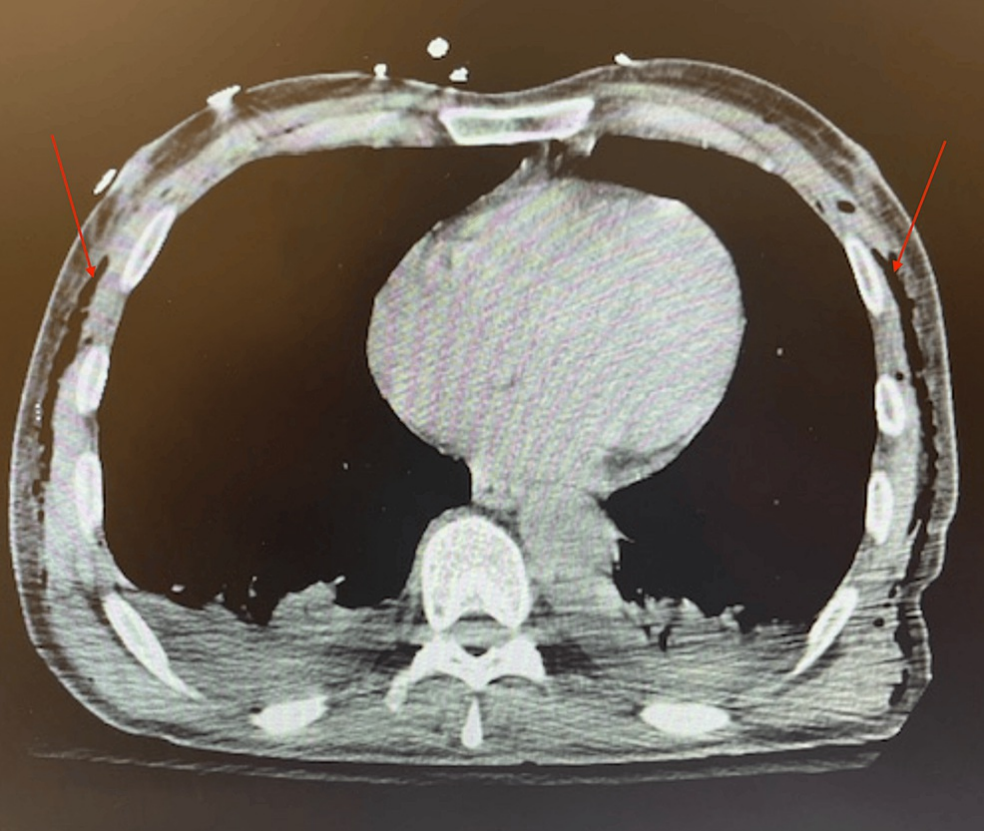
Repeat CT scan of the chest taken on postoperative day 6, which demonstrated marked improvement in subcutaneous emphysema with mild bilateral pleural effusions and bibasilar subsegmental atelectasis.

The patient remained intubated in the ICU on 100% oxygen for more than 48 hours. The only hemodynamic support required during that time was nicardipine for the management of hypertension. On postoperative day 3, the patient was extubated after confirmation of a positive endotracheal cuff leak and placed at 4 liters per minute of oxygen through a nasal cannula. The patient was lethargic but in no respiratory distress. Mental status exams improved throughout the day. A repeat CT scan of the chest was conducted on postoperative day 6 and demonstrated marked improvement in subcutaneous emphysema, with mild bilateral pleural effusions and bibasilar subsegmental atelectasis, as shown in Figure 5.

The patient was transferred from the ICU to surgical step-down on postoperative day 7. The patient remained in the hospital ward until postoperative day 17 due to the management of ileus and AKI prior to his successful discharge to home. 

## Discussion

Subcutaneous emphysema refers to the presence of air in the subcutaneous layer of the skin [1,3,4]. About 70% of cases of subcutaneous emphysema that have been reported have occurred in males, with an average age of 53 years [3]. Although severe cases of subcutaneous emphysema that result in respiratory and cardiovascular compromise are very rare (0.3-3.0%), grossly undetectable subcutaneous emphysema may occur in up to 77% of patients who undergo laparoscopic procedures [1,3,4]. The pathogenesis of subcutaneous emphysema has been attributed to multiple possible causes, such as malfunctions in ventilator circuits or pop-off valves, trauma to the airway, chest wall or bowel, or following insufflation of the abdomen during laparoscopic or robotic procedures [2]. In severe cases, complications such as delayed extubation, pneumomediastinum, pneumoperitoneum, and pneumothorax may occur due to extravasation of air into other bodily spaces from pressure gradients between anatomic and fascial planes [1,6,11]. In this case, it is our belief that the likely cause of diffuse subcutaneous emphysema was the placement of trocars and insufflation of the abdomen for the robotic total colectomy. The CT scans of the neck and chest obtained following transfer to the ICU revealed no evidence of trauma to the airway or injury to the esophagus, and there were no intraoperative surgical complications. Studies have demonstrated that the risk of subcutaneous emphysema following robotic-assisted laparoscopic surgery is greater in older patients, lengthy surgical procedures with more than five entry ports, and with the utilization of high CO_2_ insufflation pressures (e.g., 15-20 mmHg) [11]. The extent of subcutaneous emphysema is typically described as “mild,” “moderate,” or “massive,” which refers to the presence of crepitus at the trochar insertion sites, extending to the abdomen, or extending to the chest, neck, face, and extremities, respectively [11]. In robotic-assisted laparoscopic surgeries, subcutaneous emphysema usually develops due to the dissection of tissue around the trocar sites while attempting to insert or remove the ports or due to high insufflation pressures [11]. During these maneuvers, CO_2_ can diffuse into the subcutaneous space, resulting in various degrees of subcutaneous emphysema [11]. 

The diagnosis of subcutaneous emphysema often begins clinically with the palpation of crepitus on physical exam and by observing noticeable distention or bloating of the face, neck, chest, and/or abdomen [6]. Patients may also experience severe cardiovascular or respiratory compromise, especially if an airway is lost or removed prior to diagnosing the condition, emphasizing the importance of a high index of suspicion in clinical scenarios at increased risk for this condition [4]. In the patient presented in our case, the first sign of subcutaneous emphysema was the obscuration of anatomical landmarks and gross amounts of subcutaneous air on ultrasound during an attempted TAP block for postoperative pain management. Extensive crepitus on palpation of the patient’s abdomen, chest, upper and lower extremities, and neck led to the decision to leave the patient intubated and transfer him to the ICU for close monitoring. Radiographic evidence of air in the subcutaneous tissue will confirm the diagnosis of subcutaneous emphysema and determine the severity of the disease, which helps guide management in these patients [7,8]. In our patient, CT scans of the chest and neck demonstrated extensive soft tissue air in the mediastinum, thoracic walls, and neck without defects in the tracheal or esophageal walls, confirming the diagnosis. 

Management of severe subcutaneous emphysema is supportive as source control results in resolution in most patients within 10 days [9]. Some studies have demonstrated that high-concentration oxygen can help patients with severe discomfort or respiratory compromise, as this allows nitrogen washout and diffusion of gas particles [10]. Our patient remained intubated, sedated, and mechanically ventilated with 100% oxygen until postoperative day 3 when he passed an endotracheal cuff leak test and was extubated. An endotracheal cuff leak test refers to partially deflating the endotracheal tube cuff and auscultating with a stethoscope to identify an audible air leak around the cuff [12]. A negative leak test refers to the absence of an air leak and indicates an elevated risk of upper airway obstruction and re-intubation, in this case, likely due to extensive subcutaneous air [12]. A positive leak test refers to the presence of an air leak and indicates that there are likely no upper airway obstructions that would necessitate reintubation [12]. Upon extubation, the patient was mildly lethargic but was in no respiratory distress, and his mental status exams improved throughout the day. The patient maintained adequate saturations on 4L od nasal cannula and reported no discomfort. High concentration oxygen was not required and the patient was subsequently managed with close observation without additional respiratory support.

Although the patient in our case maintained appropriate levels of etCO_2_ throughout the procedure, anesthesia providers must be aware of warning signs that may indicate hypercarbia or the development of subcutaneous emphysema throughout procedures so that compensatory ventilatory adjustments can be made if necessary [11]. In contrast to the awake patient who can increase minute ventilation in response to hypercarbia, an anesthetized patient must be compensated through changes to ventilatory settings to avoid hypercarbia and acidosis [11]. A rising etCO_2_ during a robotic-assisted laparoscopic procedure may indicate subcutaneous emphysema, and insufflation should be temporarily stopped until levels decrease [11]. Anesthesia providers should remain vigilant and palpate the chest and neck of patients throughout the case, if possible, to assess for cases of severe subcutaneous emphysema.

This case report emphasizes the importance of clinicians having a high index of suspicion for the development of subcutaneous emphysema, particularly in patients undergoing laparoscopic or robotic procedures, which necessitate insufflation of the abdomen. In these cases, assessing for subcutaneous emphysema or distension in the face or neck prior to extubation should be considered to ensure the patient will be able to maintain spontaneous ventilation following extubation. In patients where there is a suspected diagnosis of severe subcutaneous emphysema, delayed extubation should be considered pending imaging studies and leak tests due to the risk of respiratory compromise and the loss of a secured airway. Further research is needed to determine which patients should remain intubated, how to identify patients at higher risk, methods of decreasing surgical risk factors (e.g., types of trocars and amount of insufflation), and intraoperative therapies to reduce the risk of developing subcutaneous emphysema.

## Conclusions

This case report describes the presentation of extensive subcutaneous emphysema following a robotic total colectomy in a 57-year-old male with a history of Lynch syndrome (hereditary nonpolyposis colorectal cancer syndrome) and left-sided colon cancer. The patient remained intubated and was transferred to the ICU for hemodynamic and respiratory monitoring with close observation of palpable crepitus. Following the improvement of subcutaneous emphysema, confirmed through physical examinations and imaging, the patient passed a leak test, was extubated, and subsequently transferred to a surgical step-down. Although severe subcutaneous emphysema such as the case presented here is rare, maintaining a high index of suspicion and conducting a careful assessment prior to extubation is important to prevent airway compromise and poor outcomes. The decision to delay extubation in these patients to prevent airway compromise must be a clinical decision guided by physical exam, patient respiratory status, and provider judgment. 
